# Supplementary material for: Fluorochromized tyramide-glucose oxidase as a multiplex fluorescent tyramide signal amplification system for histochemical analysis
Source: Sci Rep. 2022 Sep 12;12:14807. doi: 10.1038/s41598-022-19085-9 (PMC9468149; doi:10.1038/s41598-022-19085-9)
Supplement: Supplementary file 1 — Supplementary Information. [file 41598_2022_19085_MOESM1_ESM.pdf]

Supplementary Information

Title:
Fluorochromized Tyramide-Glucose Oxidase as a multiplex fluorescent tyramide signal amplification system for histochemical analysis

Kenta Yamauchi, Shinichiro Okamoto, Yoko Ishida, Kohtarou Konno, Kisara Hoshino, Takahiro Furuta, Megumu Takahashi, Masato Koike, Kaoru Isa, Masahiko Watanabe, Tadashi Isa, Hiroyuki Hioki

Supplementary Figure S1. 5-HT innervation in the mouse brain visualized with FT-GO.

Supplementary Figure S2. Quenching of Ab-conjugated POD by incubation with NaN_3.

Supplementary Table S1. Primary antibodies used in the present study.

Supplementary Table S2. Secondary antibodies used in the present study.

Supplementary Table S3. Abbreviations

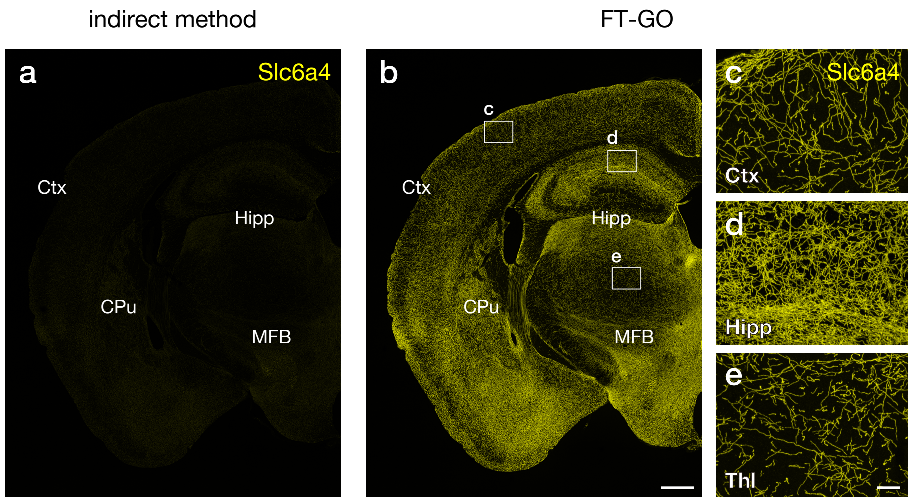

Supplementary Fig. S1| 5-HT innervation in the mouse brain visualized with FT-GO.
a, b, Slc6a4 IF in mouse brain sections visualized with an indirect (**a**) and FT-GO method (**b**) (n = 3 animals for each condition). Images are acquired with the same parameters for comparisons. Five times lower concentration of the primary Ab was applied in the FT-GO IF. **c-e.** Higher magnification images in rectangles in (**b**). CF488A tyramide is used for color development in the FT-GO method. CPu: caudate-putamen, Ctx: cerebral cortex, Hipp: hippocampus, MFB: medial forebrain bundle, Thl: thalamus. Scale bars: 500 µm in (**b**) and 50 µm in (**e**).

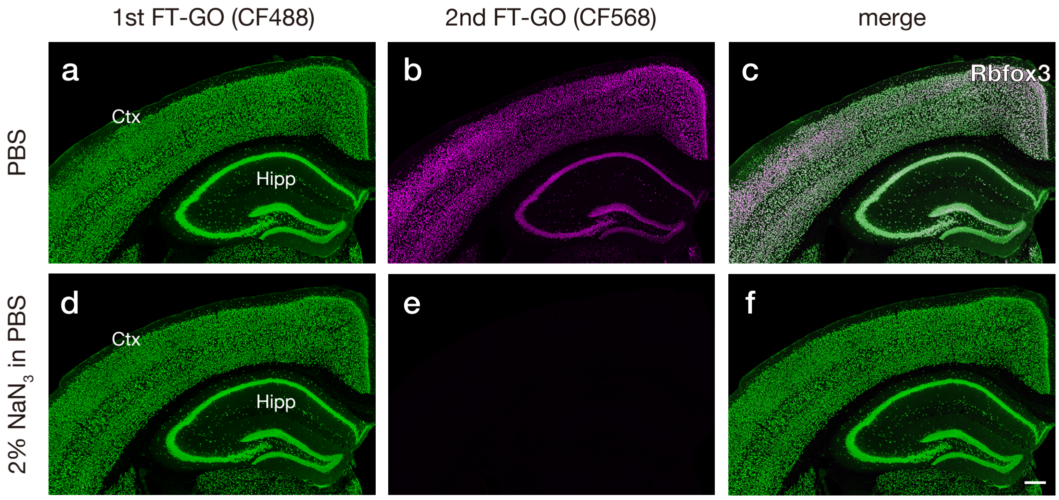

Supplementary Fig. S2| Quenching of Ab-conjugated POD by incubation with NaN_3.
a-f, Two rounds of FT-GO in IF for Rbfox3. Brain sections were treated with PBS (**a-c**) or 2% NaN_3 in PBS for 4 hr (**d-f**) between the first and second round FT-GO (n = 3 animals for each condition). First and second round of FT-GO are color-developed with CF488A (green, **a, d**) and CF568 tyramide (magenta, **b, e**). (**c**) and (**f**) show merged images of (**a**) and (**b**), and (**d**) and (**e**), respectively. Ctx: cerebral cortex, Hipp: hippocampus. Scale bar: 250 µm.

Supplementary Table S1| Primary antibodies used in the present study.

Antigen	Host species	Source, Cat. No.	RRID	Concentration or dilution
Aif1	Goat, polyclonal	FUJIFILM Wako Pure Chemical Corporation, 011-27991	n/a	1:5,000
Gfap	Rabbit, polyclonal	Sigma-Aldrich, G9269	AB_477035	1:20,000
mRFP1	Rabbit, polyclonal	Hioki et al., *J Comp Neurol*; 518, 668-686	n/a	0.1 μg/ml
proCck	Rabbit, polyclonal	Frontier Institute, CCK-pro-Rb-Af350	AB_2571674	1:500
Pvalb	Mouse, monoclonal	Sigma-Aldrich, P3088	AB_477329	1:5,000
Rbfox3	Mouse, monoclonal	Merck Millipore, MAB377	AB_2298772	1:100, 1,000, 10,000 or 100,000
Rbfox3	Mouse, monoclonal	Merck Millipore, MAB377X	AB_2149209	1:100, 1,000, 10,000 or 100,000
Slc6a4	Rabbit, polyclonal	Frontier Institute, HTT-Rb-Af560	AB_2571775	1:1,000 (indirect detection) 1:5,000 (FT-GO detection)
TH	Rabbit, polyclonal	PelFreez, P40101-150	AB_2617184	1:1,000

Supplementary Table S2| Secondary antibodies used in the present study.

Antibody	Source, Cat. No.	RRID	Concentration or dilution
CF405M Goat anti-Mouse IgG	Biotium, 20182	AB_10557262	10 µg/ml
CF488A Donkey anti-Mouse IgG	Biotium, 20014	AB_10561327	10 µg/ml
CF488A Donkey anti-Rabbit IgG	Biotium, 20015	AB_1055966	10 µg/ml
CF647 Donkey anti-Rabbit IgG	Biotium, 20047	AB_10559808	10 µg/ml
POD F(ab')2 fragment Donkey anti-Goat IgG	Jackson Immuno Research, 705-036-147	AB_2340392	1:200 or 500
POD F(ab')2 fragment Donkey anti-Mouse IgG	Jackson Immuno Research, 715-036-151	AB_2340774	1:200 or 500
POD F(ab')2 fragment Donkey anti-Rabbit IgG	Jackson Immuno Research, 711-036-152	AB_2340590	1:200 or 500

Supplementary Table S3| Abbreviations

• 5-HT	serotonergic
• AAV	adeno-associated virus
• Ab	antibody
• Amy	amygdala
• AU	arbitrary units
• BT-GO	biotinyl tyramine-glucose oxidase
• CA	catecholaminergic
• cc	corpus callosum
• Cd	caudte nucleus
• CPu	caudate-putamen
• CARD	catalyzed reporter deposition
• Ctx	cerebral cortex
• DG	dentate gyrus
• FISH	fluorescence *in situ* hybridization
• FP	fluorescent protein
• FT	fluorochromized tyramide
• FT-GO	fluorochromized tyramide-glucose oxidase
• GPe	external segment of the globus pallidus
• H_2O_2	hydrogen peroxide
• Hipp	hippocampus
• IF	immunofluorescence
• IHC	immunohistochemistry
• IR	immunoreactivity
• ISH	*in situ* hybridization
• lf	lateral fissure
• MFB	medial forebrain bundle
• NaN_3	sodium azide
• pAb	primary antibody
• PBS	phosphate buffered saline
• POD	peroxidase
• Pu	putamen
• S1	primary somatosensory cortex
• sAb	secondary antibody
• SN	substantia nigra
• sts	superior temporal sulcus
• Thl	thalamus
• TSA	tyramide signal amplification
